# Comprehensive characterization of CRC with germline mutations reveals a distinct somatic mutational landscape and elevated cancer risk in the Chinese population

**DOI:** 10.20892/j.issn.2095-3941.2021.0190

**Published:** 2022-01-12

**Authors:** Jianfei Yao, Yunhuan Zhen, Jing Fan, Yuan Gong, Yumeng Ye, Shaohua Guo, Hongyi Liu, Xiaoyun Li, Guosheng Li, Pan Yang, Xiaohui Wang, Danni Liu, Tanxiao Huang, Huiya Cao, Peisu Suo, Yuemin Li, Jingbo Yu, Lele Song

**Affiliations:** 1 Department of Radiotherapy, the Eighth Medical Center of the Chinese PLA General Hospital, Beijing 100091, China; 2 HaploX Biotechnology, Shenzhen 518057, China; 3 Department of Colorectal Surgery, Affiliated Hospital of Guizhou Medical University, Guiyang 550004, China; 4 International Business School, Beijing Foreign Studies University, Beijing 100089, China; 5 Department of Gastroenterology, the Second Medical Center of the Chinese PLA General Hospital, Beijing 100853, China; 6 Department of Experimental Pathology, Beijing Institute of Radiation Medicine, Beijing 100850, China; 7 Department of General Surgery, the First Medical Center of the Chinese PLA General Hospital, Beijing 100853, China; 8 Department of Hepatobiliary Surgery, Dalian Municipal Central Hospital, Dalian Medical University, Dalian 116033, China; 9 Comprehensive Liver Cancer Department, the Fifth Medical Center of the Chinese PLA General Hospital, Beijing 100039, China

**Keywords:** Colorectal cancer, germline, Lynch syndrome, hereditary cancer, next-generation sequencing, Notch signaling pathway, TMB, MSI, MMR

## Abstract

**Objective:**

Hereditary colorectal cancer (CRC) accounts for approximately 5%–10% of all CRC cases. The full profile of CRC-related germline mutations and the corresponding somatic mutational profile have not been fully determined in the Chinese population.

**Methods:**

We performed the first population study investigating the germline mutation status in more than 1,000 (*n* = 1,923) Chinese patients with CRC and examined their relationship with the somatic mutational landscape. Germline alterations were examined with a 58-gene next-generation sequencing panel, and somatic alterations were examined with a 605-gene panel.

**Results:**

A total of 92 pathogenic (P) mutations were identified in 85 patients, and 81 likely pathogenic (LP) germline mutations were identified in 62 patients, accounting for 7.6% (147/1,923) of all patients. MSH2 and APC was the most mutated gene in the Lynch syndrome and non-Lynch syndrome groups, respectively. Patients with P/LP mutations had a significantly higher ratio of microsatellite instability, highly deficient mismatch repair, family history of CRC, and lower age. The somatic mutational landscape revealed a significantly higher mutational frequency in the P group and a trend toward higher copy number variations in the non-P group. The Lynch syndrome group had a significantly higher mutational frequency and tumor mutational burden than the non-Lynch syndrome group. Clustering analysis revealed that the Notch signaling pathway was uniquely clustered in the Lynch syndrome group, and the MAPK and cAMP signaling pathways were uniquely clustered in the non-Lynch syndrome group. Population risk analysis indicated that the overall odds ratio was 11.13 (95% CI: 8.289–15.44) for the P group and 20.68 (95% CI: 12.89–33.18) for the LP group.

**Conclusions:**

Distinct features were revealed in Chinese patients with CRC with germline mutations. The Notch signaling pathway was uniquely clustered in the Lynch syndrome group, and the MAPK and cAMP signaling pathways were uniquely clustered in the non-Lynch syndrome group. Patients with P/LP germline mutations exhibited higher CRC risk.

## Introduction

Colorectal cancer (CRC) is the third and second most common cancer in men and women worldwide, respectively^1^, and the fifth most common cancer in China^2^. Although most cases of CRC are sporadic, inherited factors are known to contribute to approximately 30%–35% of CRC cases^3^. Approximately 5%–10% of patients with CRC carry high-risk germline mutations that are associated with known hereditary CRC syndromes, including Lynch syndrome (also known as hereditary non-polyposis CRC), familial adenomatous polyposis (FAP), MUTYH-associated polyposis, Peutz-Jeghers syndrome, juvenile polyposis syndrome, PTEN hamartoma tumor syndrome, and serrated polyposis syndrome^4–6^. The germline mutations associated with these syndromes have been extensively investigated at both the genomic and individual gene levels, and the heritability of many of these mutations has been confirmed in population and/or family studies. New germline mutations with suspected heritability have also been reported in recent years^7,8^. Many hotspot mutations have been identified in hereditary CRC syndromes, primarily involving APC, MLH1, MSH2, MSH6, and PMS2^7,8^. Therefore, hereditary CRC syndromes are associated with both hotspot and non-hotspot germline mutations.

Previous research has shown that pathogenic germline mutations increase the risk of cancers, including not only CRC^7^ but also hereditary breast and ovarian cancer syndrome^9^ and lung cancer^10^. However, this risk remains to be clearly defined for Chinese patients with CRC. Furthermore, the somatic mutational landscape of hereditary CRC syndromes has yet to be characterized and compared with that of sporadic CRC. This comparison may aid in understanding the mechanisms underlying hereditary CRC syndromes. In this study, we recruited a large cohort of 1,923 unselected patients with CRC, investigated both the germline and somatic mutational landscapes, and performed extensive comparisons between patients with and without pathogenic germline mutations. More importantly, by comparing the incidence of individual mutations in our cohort with that in the general population, we clarified the risk associated with the identified germline mutations. This study provides important information regarding the mutational landscape, cancer risk, and potential carcinogenic mechanisms of CRC-related germline mutations in the Chinese population. Our findings may help establish preventive and therapeutic strategies for patients with CRC with suspected heritability.

## Materials and methods

### Ethics approval

All experimental plans and protocols for the study were submitted to the ethics/licensing committees of the indicated participating hospitals for review and approval before the start of the clinical study, and were approved by the corresponding committees of the participating hospitals (Approval No. S2015-032-02). Because the study had a retrospective design and used retrospective samples collected by the participating hospitals, informed consent was not required. Patients with pathogenic (P) or likely pathogenic (LP) germline mutations were informed of the test results. All experiments, methods, procedures, and personnel training were carried out in accordance with the relevant guidelines and regulations of the participating hospitals and laboratories.

### Study design

The study was designed and implemented in 7 Chinese hospitals, and both cancer tissue and blood samples were collected retrospectively. The study was designed to include as many patients with CRC as possible, provided that the tissue or blood samples were available for next-generation sequencing (NGS). Samples collected between January 2016 and August 2020 from 1,923 patients with CRC were obtained according to the availability of samples for NGS testing in the participating hospitals. The details of patient demographic information, pathological information, family history, and microsatellite instability (MSI)/mismatch repair (MMR) information are summarized in **Table 1**. Family history was defined as confirmed CRC patients with at least one immediate family member (first degree relative) with a history of CRC diagnosis. The immediate family members included parents, siblings, and children. The collected samples comprised tissue samples [formalin-fixed paraffin-embedded (FFPE) samples or frozen samples from surgery] and blood samples obtained at the time of CRC diagnosis confirmation. Diagnosis was confirmed with imaging examinations and subsequent pathological examinations. No participants received chemotherapy, radiotherapy, targeted therapy, or immunotherapy before the tissue and blood samples were collected. The somatic sequencing data presented in this study were from FFPE samples or frozen tissue samples. Germline sequencing data were obtained from the corresponding genomic DNA of white blood cells.

**Table 1:** Demographic information and MSI/MMR status for recruited patients

	Total (*n* = 1,923)	%	P (*n* = 85)	%	LP (*n* = 62)	%	non-P (*n* = 1,776)	%	*P* (P *vs.* non-P)	*P* (LP *vs.* non-P)
Stage									0.13	0.00032
I	183	0.095	2	0.024	1	0.016	180	0.101		
II	833	0.433	39	0.459	37	0.597	757	0.426		
III	527	0.274	25	0.294	6	0.097	496	0.279		
IV	380	0.198	19	0.224	18	0.29	343	0.193		
Age									0.001	0
<40	182	0.095	16	0.188	4	0.065	162	0.091		
40–49	332	0.173	21	0.247	25	0.403	286	0.161		
50–59	511	0.266	18	0.212	14	0.226	479	0.270		
≥60	810	0.421	25	0.294	15	0.242	770	0.434		
NA	88	0.046	5	0.059	4	0.065	79	0.044		
Gender									0.458	0.288
Male	1130	0.588	52	0.612	33	0.532	1045	0.588		
Female	728	0.379	28	0.329	28	0.452	672	0.378		
NA	65	0.034	5	0.059	1	0.016	59	0.033		
Family history									0.014	0.026
Yes	111	0.058	8	0.094	7	0.113	96	0.054		
No	734	0.382	21	0.247	19	0.306	694	0.391		
NA	1078	0.561	56	0.659	36	0.581	986	0.555		
MSI status									0	0
MSI-H	113	0.059	25	0.294	18	0.290	68	0.038		
MSI-L	21	0.011	2	0.024	2	0.032	17	0.010		
MSS	1577	0.820	47	0.553	38	0.613	1492	0.840		
NA	214	0.111	11	0.129	4	0.065	199	0.112		
MMR status									0	0
dMMR	82	0.043	19	0.224	15	0.242	48	0.027		
pMMR	750	0.390	27	0.318	11	0.177	712	0.401		
NA	1091	0.567	39	0.459	36	0.581	1016	0.572		

P, pathogenic; LP, likely pathogenic; non-P, non-pathogenic; MSI, microsatellite instability; MSI-H, microsatellite instability high; MSI-L, microsatellite instability low; MSS, macrosatellite stable; MMR, mismatch repair; dMMR, deficient mismatch repair; pMMR, proficient mismatch repair; NA, not available.

### Sample preparation, targeted NGS, and data processing

For the FFPE samples, ten 5 μm tumor slices were used for DNA extraction with a QIAamp DNA FFPE Kit (QIAGEN, Valencia, CA, USA) according to the manufacturer’s instructions. For tissue samples, a minimum of 50 mg tissue was used for DNA extraction with a QIAamp DNA Mini Kit (QIAGEN, Valencia, CA, USA). For blood samples, 2 mL of blood was collected in tubes containing EDTA and centrifuged at 1,600 × g for 10 min at 4 °C within 2 h of collection. The peripheral blood lymphocyte (PBL) debris was stored at −20 °C until further use. DNA from PBLs was extracted with a RelaxGene Blood DNA system (Tiangen Biotech Co., Ltd., Beijing, China) according to the manufacturer’s instructions. Both cancer tissue and white blood cell genomic DNA were quantified with a Qubit 2.0 fluorometer and Qubit dsDNA HS assay kit (Thermo Fisher Scientific, Inc., Waltham, MA, USA) according to the manufacturer’s instructions. Fragmented genomic DNA underwent end-repair, A-tailing, and ligation with indexed adapters sequentially, followed by size selection with Agencourt AMPure XP beads (Beckman Coulter Inc., Brea, CA, USA). DNA fragments were used for library construction with a KAPA Library Preparation kit (Kapa Biosystems, Inc., Wilmington, MA, USA) according to the manufacturer’s protocol. Hybridization-based target enrichment was performed with a HaploX germline gene panel (58 known hereditary cancer-related genes, HaploX Biotechnology; gene list in **Supplementary Table S1**) for white blood cell genomic DNA or a HaploX pan-cancer gene panel (605 cancer-relevant genes, HaploX Biotechnology; gene list in **Supplementary Table S2**) for cancer tissue sequencing. Depending on the amount of DNA used, 7 to 8 polymerase chain reaction cycles were performed with pre-capture ligation-mediated polymerase chain reaction oligonucleotides (Kapa Biosystems, Inc.) in 50 μL reactions. DNA sequencing was then performed on an Illumina Novaseq 6000 system according to the manufacturer’s recommendations at an average depth of 2,200× for tissue and FFPE samples.

Data meeting the following criteria were included in subsequent analysis: ratio of remaining data filtered by fastq in raw data ≥85%; proportion of Q30 bases ≥85%; ratio of reads on the reference genome ≥85%; target region coverage ≥98%; and average sequencing depth in tissues ≥2,200×. The called somatic variants were required to meet the following criteria: read depth at a position ≥20×; variant allele fraction (VAF) ≥2% for tissue and PBL genomic DNA; somatic-*P* value ≤0.01; strand filter ≥1. VAF values were calculated for Q30 bases. The copy number variation (CNV) was detected with CNVkit version 0.9.3 (https://github.com/etal/cnvkit). Further analyses of genomic alterations were also performed, including single nucleotide variants (SNVs), insertion/deletion (indels), and CNVs.

### Interpretation of pathogenicity of germline mutations and calculation of somatic TMB

The pathogenicity of germline mutations was defined and predicted according to the 5-grade classification system of the American College of Medical Genetics and Genomics Guidelines for the Interpretation of Sequence. All germline mutations were categorized into P, LP, or non-pathogenic (non-P) groups. The variants of uncertain significance (VUS), and benign and likely benign mutations were defined as the non-P group in this study. TMB was calculated by division of the total number of tissue non-synonymous SNP and indel variations (VAF > 2%) by the full length of the exome region of the 605-gene NGS panel (**Supplementary Table S2**). The genomic sequence from the DNA of PBLs was used for genomic alignment when calling the somatic mutations.

### Statistical analysis

Statistical analysis was performed, and figures were plotted in GraphPad Prism 5.0 software (GraphPad Software, Inc, La Jolla, CA, USA). Student’s t-test was performed when 2 groups were compared, and analysis of variance and post hoc tests were performed when 3 or more groups were compared. Chi-square test and Fisher’s test were performed when rates or percentages were compared for significance. Figures for the mutation spectrum were produced with R software (https://www.r-project.org/). Data for pathway enrichment analysis were analyzed with the method described by DAVID Bioinformatics Resources 6.8 (https://david.ncifcrf.gov/) and were visualized with corresponding packages for R software. The protein-protein interaction network was analyzed with the STRING database, and the hub genes were determined with Cytoscape software (cytoscape.org); the Degree method was used to rank the genes. The odds ratio (OR) was calculated on the basis of the frequency of a certain germline mutation from the Genome Aggregation Database (gnomAD) in the general population and the corresponding mutation frequency obtained from this study. The OR and 95% confidence interval (CI) for each germline mutation was calculated in SPSS 17.0 software (IBM China Company Limited, Beijing, China). *P < 0.05; **P < 0.01; and ***P < 0.001.

## Results

### The panorama of germline mutations in Chinese patients with CRC

**Figure 1:**
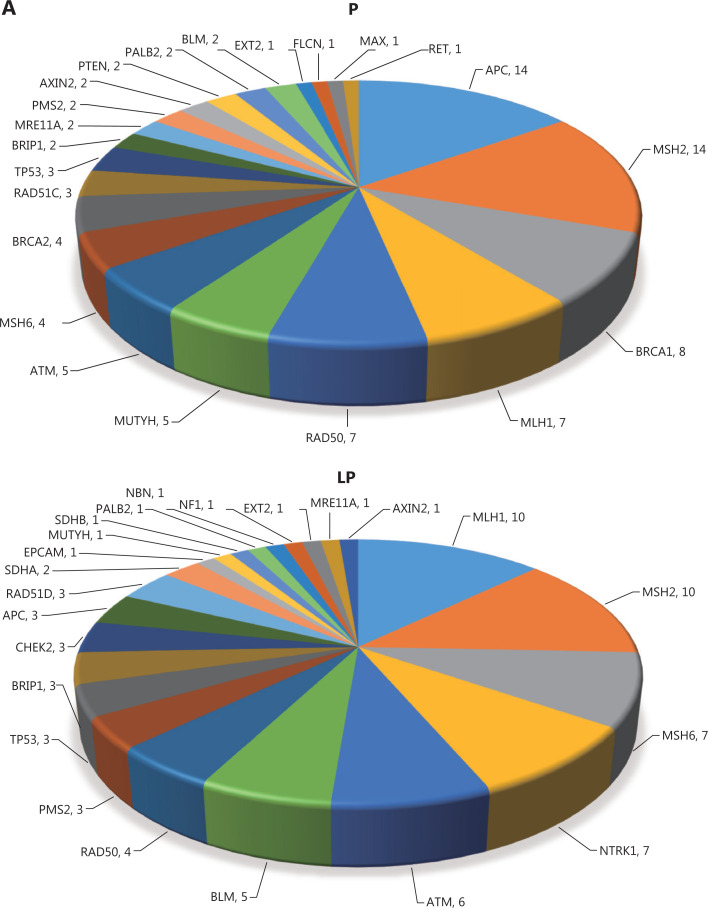

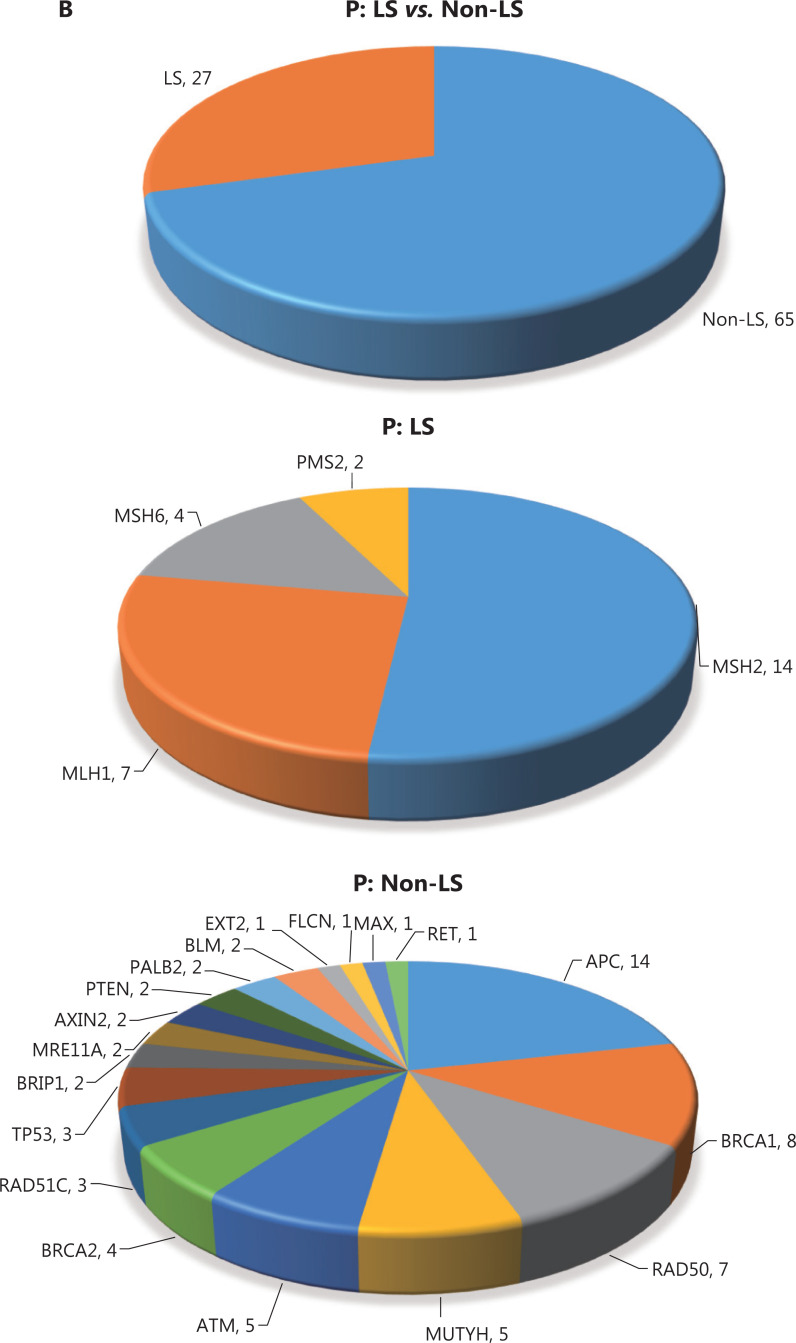
Category and distribution of germline mutations in the Chinese population. A. The number of mutations in highly mutated genes in the pathogenic (P) and likely pathogenic (LP) groups. B. Details of mutated genes and their numbers in the Lynch syndrome (LS) and non-Lynch syndrome (non-LS) groups.

First, we investigated the genetic landscape of germline alterations in all 1,923 recruited patients with CRC, among whom we identified 92 P germline mutations in 85 patients (**Figure 1A**) and 81 LP germline mutations in 62 patients (**Figure 1A**). The remaining 1,776 patients carried VUS, benign, or likely benign germline alterations (non-P). The proportion of patients with P or LP germline mutations was 7.6% (147/1,923). The highest number of P mutations was seen in APC and MSH2 (*n* = 14), followed by BRCA1 (*n* = 8), MLH1 (*n* = 7), and RAD50 (*n* = 7). MLH1 and MSH2 exhibited the highest number of LP mutations (*n* = 10), followed by MSH6 (*n* = 7), NTRK1 (*n* = 7), and ATM (*n* = 6). Further analysis indicated that 27 of 92 P mutations were detected in patients who had been diagnosed with Lynch syndrome (**Figure 1B, left panel**). MSH2 was the gene associated with the most mutations in Lynch syndrome (14) and was followed by MLH1 (*n* = 7), MSH6 (*n* = 4), and PMS2 (*n* = 2) (**Figure 1B, middle panel**). For patients without Lynch syndrome, APC was identified as the gene associated with the most mutations (*n* = 14) and was followed by BRCA1 (*n* = 8), RAD50 (*n* = 7), MUTYH (*n* = 5), ATM (*n* = 5), and BRCA2 (*n* = 4) (**Figure 1B, right panel**).

Interestingly, we observed a significantly higher ratio of patients with MSI-H or dMMR in the P or LP group than the non-P group (**Table 1**). We also identified a significantly higher ratio of patients with family history in the P and LP groups than the non-P group. Patients with P or LP mutations were significantly younger than those in the non-P group (**Table 1**). A significant difference in stage distribution was observed between the LP and the non-P group, possibly because of the low number of patients in the LP group in stages I and III. We observed no significant differences in P and LP germline mutations between males and females (**Table 1**).

**Figure 2:**
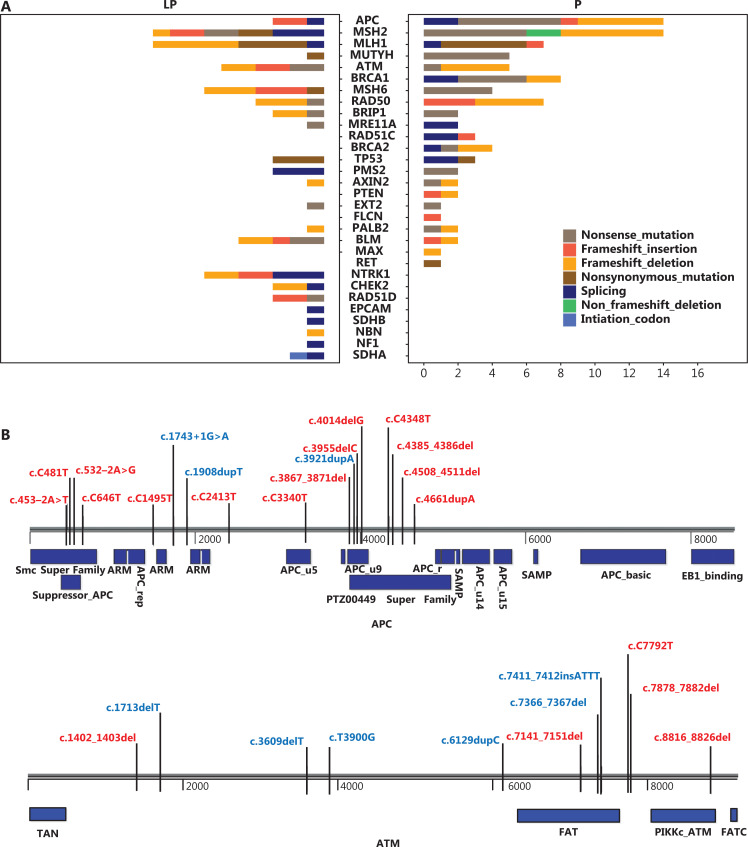

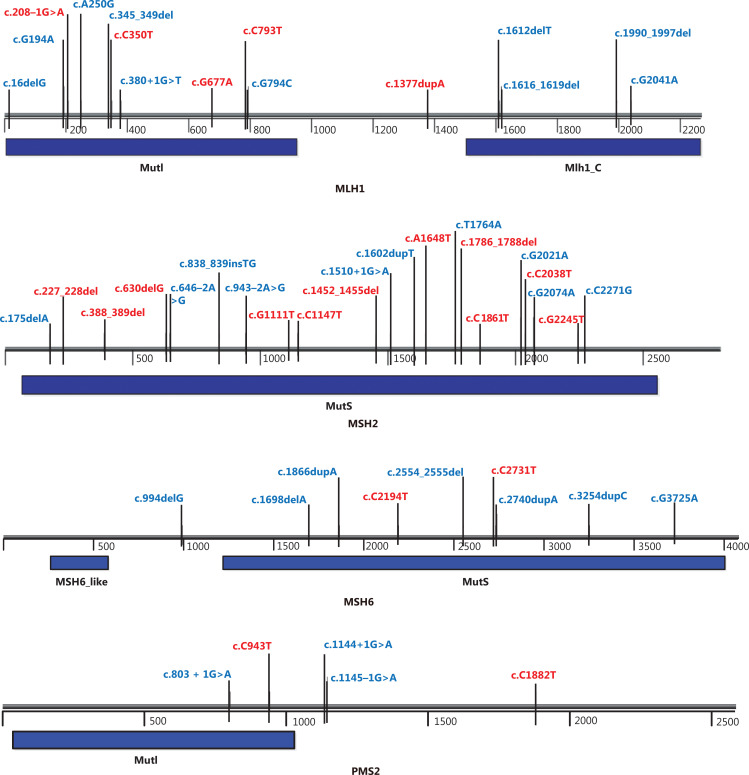
Types and distribution of mutations in highly mutated genes. A. Types and numbers of germline mutations in the P and LP groups. B. Distribution of P (red) and LP (blue) mutations in highly mutated genes, including APC, ATM, MLH1, MSH2, MSH6, and PMS2. Blue bars indicate key functional domains.

Next, we identified the specific types of mutations related to the P and LP alterations. Most mutations involved frameshift (deletion and insertion), nonsense, nonsynonymous (single nucleotide mutations), or splicing (**Figure 2A**). These mutations may cause large fragment changes or key amino acid alterations in proteins and therefore substantially influence gene function and potentially lead to high susceptibility to CRC. APC, MSH2, and MLH1, identified as the 3 genes with the highest number of P and LP mutations, might lead to familial adenomatous polyposis and Lynch syndrome.

The distribution of germline mutations in the highly mutated genes is shown in **Figure 2B**. Both P (red) and LP (blue) mutations of APC, ATM, MLH1, MSH2, MSH6, and PMS2 are plotted on individual gene schemes. Most germline mutations were located in key functional domains (blue bars). This effect was most prominent for APC, in which several mutations were distributed in the suppressor APC, APC_u9, and PTZ00449 superfamily domains. This observation suggested that P/LP germline mutations within key functional domains are more likely to be pathogenic than other mutations.

We identified several novel, previously unreported germline mutations in the dbSNP, gnomAD, and ClinVar databases (**Table 2**). These mutations included frameshift, nonsense, and splicing mutations potentially causing large fragment alterations in genes. All were classified as LP mutations, owing to their deleterious properties and undetermined clinical significance. Interestingly, patients with mismatch repair-related gene mutations (MSH2 and MSH6) and NTRK1 germline mutations exhibited very high levels of somatic TMB and a high ratio of MSI-H, thus suggesting that these mutations might behave in the same manner as known P mutations, although further clinical evidence is needed to validate this hypothesis.

**Table 2:** Novel mutations identified in this study

Gene symbol	Nucleotide change	Protein change	Mutation type	TMB	MSI status
APC	c.3921dupA	p.I1307fs	Frameshift insertion	1.63	MSS
APC	c.1908dupT	p.G636fs	Frameshift insertion	3.45	MSS
ATM	c.1713delT	p.S571fs	Frameshift deletion	2.3	MSS
ATM	c.7411_7412insATTT	p.I2471fs	Frameshift insertion	34.05	MSI-H
ATM	c.T3900G	p.Y1300X	Nonsense mutation	5.34	MSS
ATM	c.7366_7367del	p.K2456fs	Frameshift deletion	2.63	MSS
ATM	c.6129dupC	p.G2043fs	Frameshift insertion	3.38	MSS
BLM	c.C3678A	p.C1226X	Nonsense mutation	0.68	MSS
BLM	c.1440dupT	p.S480fs	Frameshift insertion	0.63	MSS
BLM	c.3354delC	p.F1118fs	Frameshift deletion	3.28	MSS
BRIP1	c.C1471T	p.Q491X	Nonsense mutation	0.88	MSS
EXT2	c.C174G	p.Y58X	Nonsense mutation	3.05	MSS
MRE11A	c.929_930insTGATTAGCTAGAACAATATCCTCCATGAAAAAC TGCCGCACTGTGTGAAGAGGAATTTTATGCATATTCATCTTCA CACAGTGCGGCAGTTTTTCATGGA	p.E310_D311delinsDDX	Nonsense mutation	2.23	MSS
MSH2	c.838_839insTG	p.L280fs	Frameshift insertion	47.33	MSI-H
MSH2	c.175delA	p.K59fs	Frameshift deletion	23.33	MSI-L
MSH2	c.1602dupT	p.R534fs	Frameshift insertion	14.98	MSS
MSH2	c.T1764A	p.Y588X	Nonsense mutation	51.4	MSI-H
MSH2	c.C2271G	p.Y757X	Nonsense mutation	1.1	MSI-H
MSH6	c.2554_2555del	p.K852fs	Frameshift deletion	36.64	MSI-H
MSH6	c.1866dupA	p.I622fs	Frameshift insertion	36.43	MSI-H
MSH6	c.1698delA	p.G566fs	Frameshift deletion	54.73	NA
MSH6	c.994delG	p.E332fs	Frameshift deletion	2.18	MSS
MSH6	c.2740dupA	p.D913fs	Frameshift insertion	43.38	MSI-H
NBN	c.1651delA	p.R551fs	Frameshift deletion	25.23	MSS
NF1	c.3198-1G>T	NA	Splicing	3.25	NA
NTRK1	c.474_475del	p.W158fs	Frameshift deletion	34.05	MSI-H
NTRK1	c.477_478insGC	p.L159fs	Frameshift insertion	34.05	MSI-H
NTRK1	c.477_478insGC	p.L159fs	Frameshift insertion	25.23	MSS
NTRK1	c.474_475del	p.W158fs	Frameshift deletion	25.23	MSS
PALB2	c.1400delG	p.G467fs	Frameshift deletion	5.34	MSS
PMS2	c.1145-1G>A	NA	Splicing	0.65	MSS
RAD50	c.887delT	p.V296fs	Frameshift deletion	5.83	MSS
RAD50	c.134delT	p.I45fs	Frameshift deletion	10.69	MSS
RAD51D	c.627dup	p.A210Cfs*114	Frameshift insertion	18.08	MSS
SDHA	c.1064+2T>C	NA	Splicing	2.58	MSS

### Correlations among characteristic somatic mutational landscapes, functional alterations, and germline mutations in CRC

**Figure 3:**
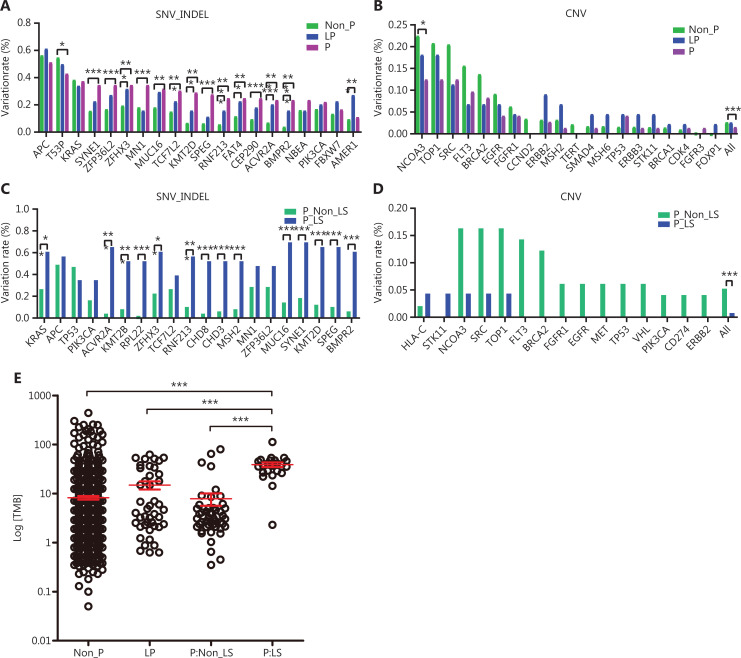
Comparison of somatic mutational frequency of highly mutated genes among the P, LP, and non-P groups. A. Comparison of somatic SNV/indel frequency among groups. B. Comparison of somatic CNV frequency among groups. C. Comparison of somatic SNV/indel frequency between patients with and without Lynch-related P germline mutations. D. Comparison of somatic CNV frequency between patients with and without Lynch-related P germline mutations. E. Comparison of TMB among the P, LP, and non-P groups. *P < 0.05; **P < 0.01; ***P < 0.001.

**Figure 4:**
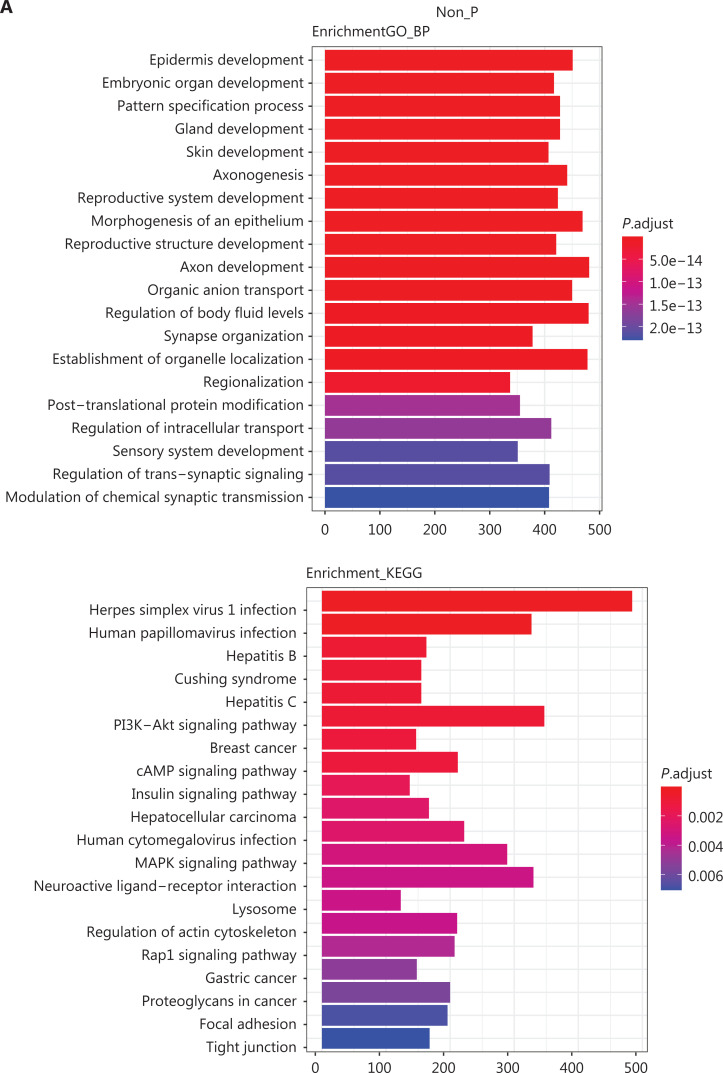

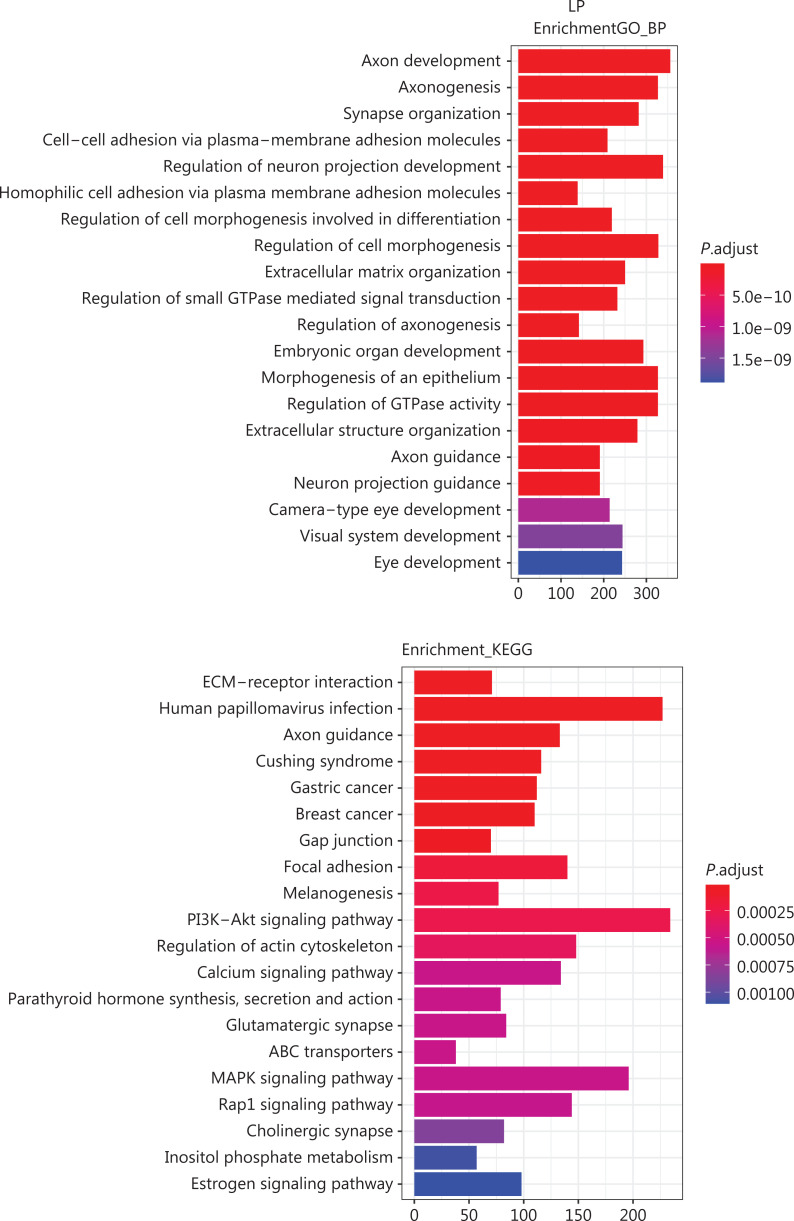

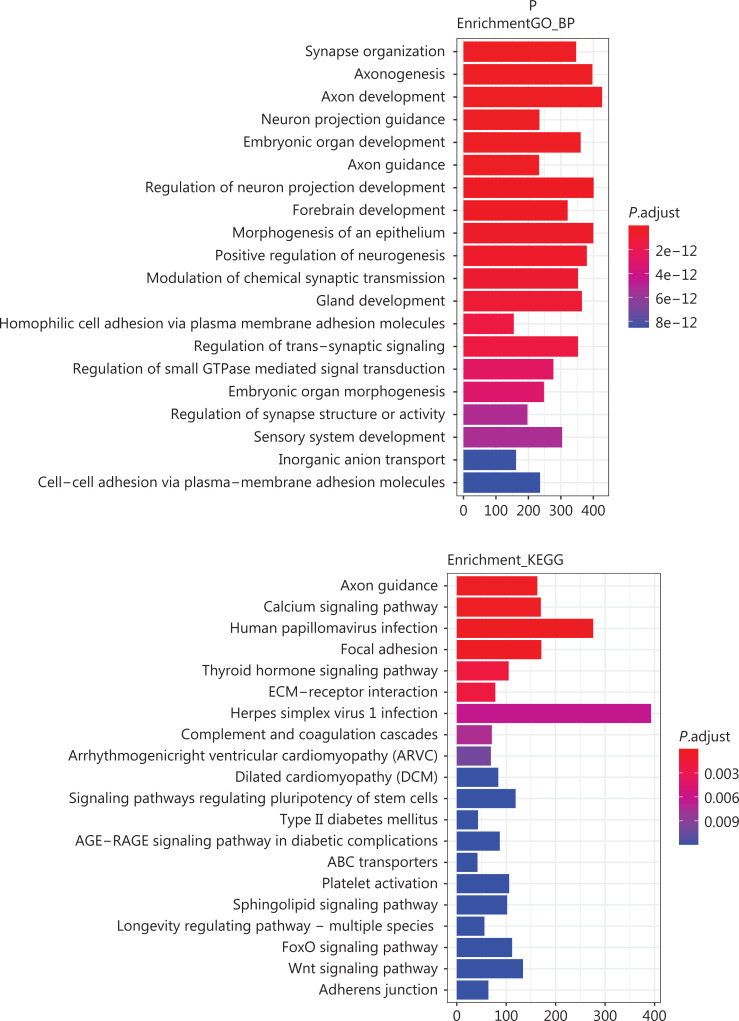

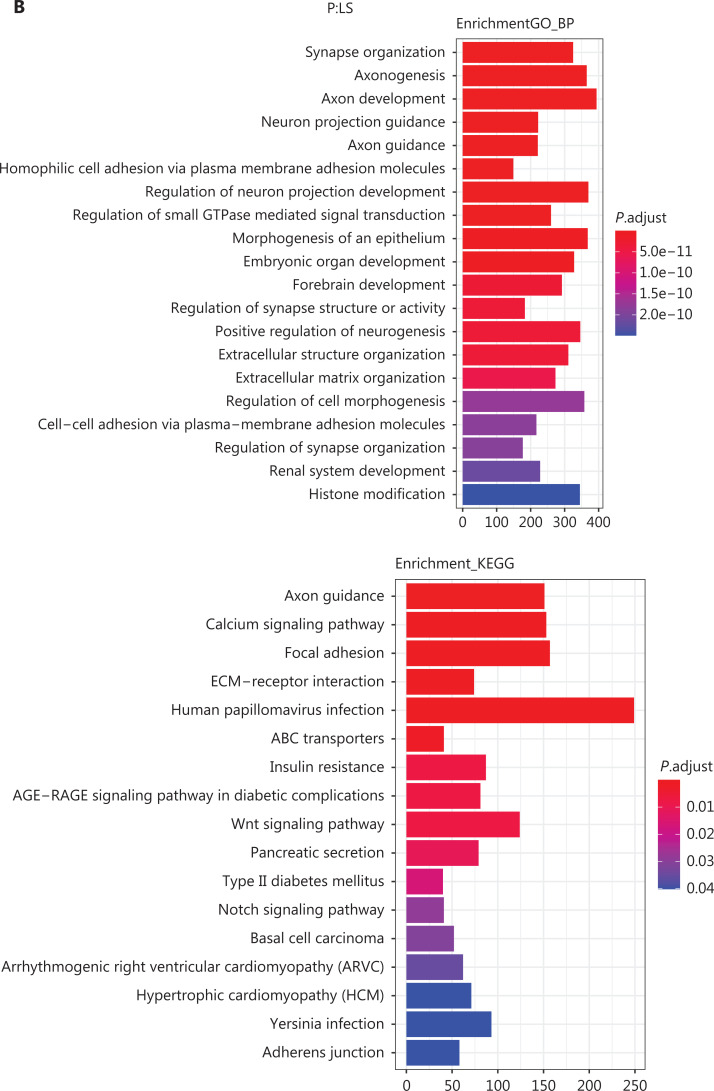

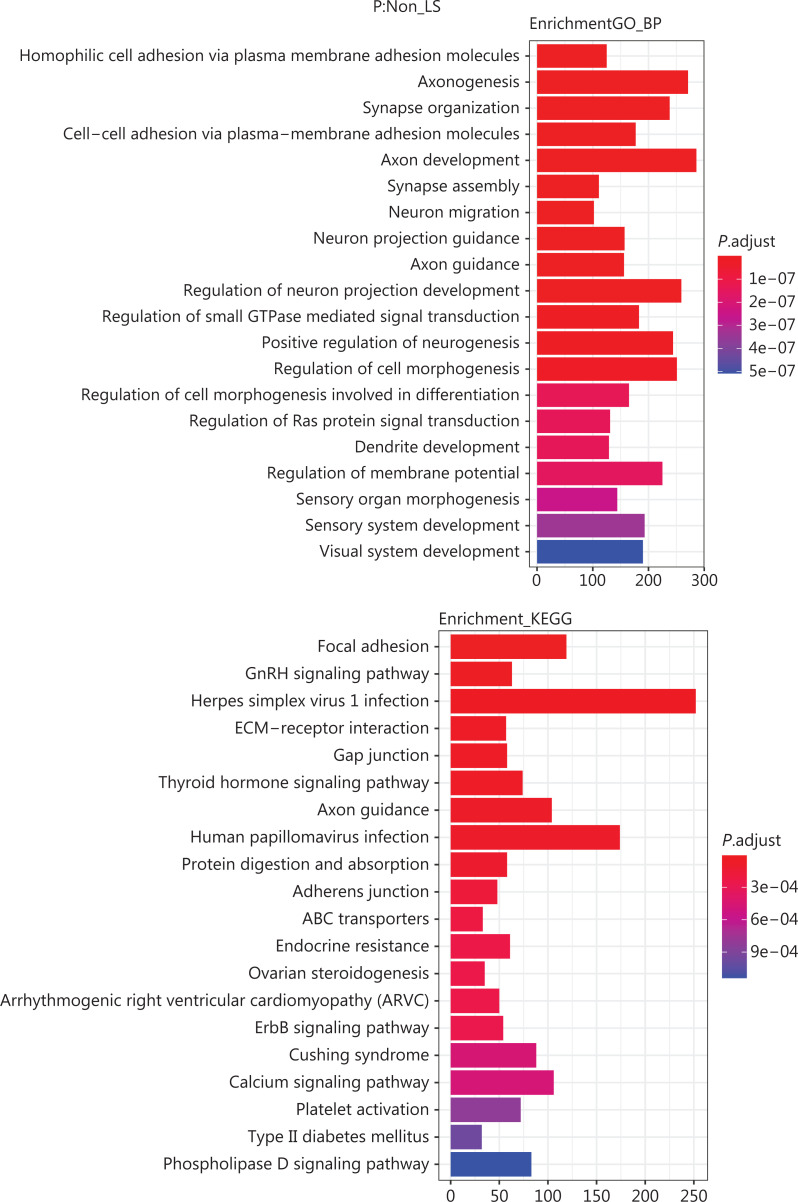
Representative highly significant somatic pathway clustering for the P, LP, and non-P groups. A. GO (biological function, BP) and KEGG somatic pathway clustering results for the groups. B. GO (BP) and KEGG somatic pathway clustering results for patients with or without Lynch P germline mutations.

The somatic mutational features of CRC with germline mutations, and how this condition relates to sporadic CRC, remain to be investigated in detail. Here we studied the somatic mutational features of CRC with or without P/LP germline mutations (**Supplementary Figure S1A**), focusing specifically on the differences among the P, LP, and non-P groups in terms of individual gene mutational frequency (**Figure 3A–D**), TMB (**Figure 3E**), and mutations significantly affecting pathways or functions (**Figure 4**).

We identified substantial differences in the SNV/indel mutational frequency of highly mutated genes (**Figure 3A**). For many genes, including TP53, SYNE1, and KMT2D, a significantly higher mutational frequency was identified in the P group than the non-P group. Similarly, a higher mutational frequency was found in the LP group than the non-P group in several genes, including ZFHX3 and KMT2D. Interestingly, the mutational frequency of APC and KARS did not differ among the 3 groups. In contrast, most CNV alterations did not differ significantly across the 3 groups, except for NCOA3 (*P* < 0.05), although we did observe a trend toward higher CNV alterations in the non-P group (**Figure 3B**). The overall CNV rate of the P group was significantly lower than that of the non-P group (*P* < 0.001).

Next, we investigated the difference between the Lynch syndrome and non-Lynch syndrome groups with P mutations (**Supplementary Figure S1B**). Patients with Lynch syndrome exhibited a significantly higher mutational frequency than those who did not have Lynch syndrome (**Figure 3C**); this was the case for most genes, except APC, TP53, and PIK3CA, whose mutational frequency did not significantly differ. In contrast, patients without Lynch syndrome exhibited a trend toward a higher frequency of CNV alterations than those with Lynch syndrome, although this association was not significant (**Figure 3D**). Next, we examined and compared the TMB for the P (including both patients with and without Lynch syndrome), LP, and non-P groups. Patients with Lynch syndrome and P mutations exhibited a much higher TMB than patients without Lynch syndrome with P mutations, and patients from the LP and non-P groups (**Figure 3E**).

To further investigate the similarities and differences in somatic mutations among the P, LP, and non-P groups, and to study the mechanistic discrepancies between Lynch syndrome and patients without Lynch syndrome with CRC, we performed gene ontology (GO) and Kyoto Encyclopedia of Genes and Genomes (KEGG) clustering analysis and compared the results from each group. **Figure 4A** shows the most significant clustering in the GO (upper row) and KEGG (lower row) analysis for the P, LP, and non-P groups. Some common biological processes, functions, and pathways were observed among the groups, together with several substantial differences. The common clustering for GO and KEGG findings across the 3 groups is summarized in **Supplementary Table S3**. Although the 3 groups of patients had distinct hereditary backgrounds, they shared several common aberrant pathways, thus potentially indicating common carcinogenic mechanisms, including the Wnt signaling pathway, calcium signaling pathway, MAPK signaling pathway, cAMP signaling pathway, and human papillomavirus infection. In contrast, we observed distinct differences between the P/LP groups and the non-P group in terms of biological processes, functions, and pathways, as shown in **Supplementary Table S4** (GO clustering) and **Supplementary Table S5** (KEGG clustering). Notably, the Notch signaling pathway was clustered in the P/LP groups but not the non-P group (**Supplementary Table S5**).

Similarities and differences were also compared between the Lynch syndrome and non-Lynch syndrome groups with regard to P germline mutations. **Figure 4B** shows the most significant clustering in GO (upper row) and KEGG (lower row) analysis for the Lynch syndrome and non-Lynch syndrome groups. Common clustering is shown in **Supplementary Table S6**. The most common pathways were the Wnt signaling pathway, the calcium signaling pathway, and human papillomavirus infection. Differences in the biological processes in terms of GO clustering are listed in **Supplementary Table S7**; interestingly, a large amount of Lynch-unique clustering was observed. Differences in KEGG clustering are shown in **Supplementary Table S8**. Notably, the Notch signaling pathway was clustered in the Lynch syndrome group but not the non-Lynch syndrome group, whereas the MAPK signaling pathway and AMP signaling pathway were clustered in the non-Lynch syndrome group but not the Lynch syndrome group. Information related to the genes enriched in each GO and KEGG category in **Figure 4** is provided in **Supplementary Table S9** (GO enrichment) and **Supplementary Table S10** (KEGG enrichment).

Next, we used the STRING database to analyze the protein interaction network for each subgroup. The top 20 genes in terms of protein interaction are listed in **Supplementary Table S11**. Each subgroup was compared with the P group, and the same genes are labeled with identical colors. In all groups, TP53 was the most common interacting gene. However, EGFR and SRC genes were found in the LP, non-P, and non-Lynch syndrome groups, but not in the P group, thus suggesting substantial differences in the protein interaction network. NOTCH1 was found only in the P and P-Lynch syndrome groups but not in the other groups, thus verifying the results of the pathway enrichment analysis. These findings strongly suggest that the mechanism of carcinogenesis in patients with P germline mutations is distinct from that in patients with no P germline mutations.

### Germline mutations increase the risk of CRC in the Chinese population

P or LP germline mutations may increase cancer susceptibility and risk. To quantify the risk of CRC in individuals carrying P or LP germline mutations, we calculated the ORs for individual germline mutations and all mutations as a whole. The prevalence of all germline mutations in the general population was determined by gnomAD screening. By comparing the prevalence in the general population and the mutation frequency identified in this study, we calculated the OR for each mutation site, or all mutations as a whole, as an indicator of CRC risk. **Table 3** shows the detailed demographic information, gene names, variation sites, allele counts, allele frequencies in the general population, and ORs for each P germline mutation detected in this study. The overall OR for all P mutations was 11.13 (95% CI:8.289–15.44). Similarly, **Table 4** shows demographic and mutational information, along with the calculated OR of all LP mutations, with an overall OR of 20.68 (95% CI: 12.89–33.18). These results indicated strong enrichment in P or LP mutations in the studied population of patients with CRC, thus indicating a significantly higher risk of CRC in patients carrying these germline mutations.

**Table 3:** Pathogenic germline mutations identified in this study

Patient ID	Age, years	Gender	Gene symbol	Nucleotide change	Allele count in this study	Allele frequency in general population*	OR	95% CI	Annotation
1	73	Male	BRCA2	c.C3109T	1	4.09216E-06	63.56	3.975–1016	P
2	48	Male	BRIP1	c.C1066T	1	8.12704E-06	32	2.901–353.0	P
3	66	Male	RAD50	c.2157dupA	3	0.000268067	2.911	0.9139–9.274	P
4	56	Male							
5	50	Male							
6	NA	NA	APC	c.4508_4511del	1	.	NA	NA	P
7	64	Female	RAD51C	c.390dupA	1	3.70044E-05	7.028	0.8902–55.49	P
8	53	Male	AXIN2	c.C1966T	1	NA	NA	NA	P
9	52	Male	MLH1	c.C793T	1	NA	NA	NA	P
10	46	Male	MSH2	c.C1147T	1	NA	NA	NA	P
11	84	Male	MRE11A	c.659+1G>A	1	2.03676E-05	12.77	1.491–109.3	P
12	22	Male	MSH2	c.C2038T	1	4.06147E-06	64.04	4.005–1024	P
13	65	Female	RAD51C	c.905-2A>C	2	8.12566E-06	64.03	9.017–454.7	P
14	44	Female							
15	25	Female	MSH2	c.1786_1788del	2	4.06105E-06	128.1	11.61–1413	P
16	NA	NA							
17	37	Male	MSH2	c.C1861T	1	NA	NA	NA	P
18	63	Female	BRCA1	c.4186-2A>G	1	NA	NA	NA	P
19	76	Female	ATM	c.7878_7882del	1	4.07176E-06	63.87	3.994–1021	P
20	54	Male	BRCA2	c.7976+1G>A	1	NA	NA	NA	P
21	46	Male	APC	c.C1495T	1	NA	NA	NA	P
22	47	Male	PTEN	c.963delA	1	NA	NA	NA	P
22	47	Male	EXT2	c.C166T	1	1.21849E-05	21.34	2.220–205.2	P
22	47	Male	APC	c.C4348T	1	0	191.9	7.815–4710	P
22	47	Male	APC	c.4385_4386del	1	NA	NA	NA	P
22	47	Male	BRCA1	c.C4327T	1	2.43756E-05	10.67	1.284–88.64	P
22	47	Male	BRCA2	c.9090delA	1	1.24512E-05	20.89	2.172–200.8	P
23	21	Female	APC	c.532-2A>G	1	NA	NA	NA	P
24	43	Male	MLH1	c.C350T	2	4.06246E-06	128.1	11.61–1413	P
25	33	Male							
26	32	Male	MSH2	c.388_389del	1	NA	NA	NA	P
27	71	Male	BRCA2	c.3854delA	1	0.000015582	16.69	1.736–160.5	P
28	65	Male	MSH2	c.1452_1455del	3	NA	NA	NA	P
29	30	Female							
30	61	Female							
31	35	Male	MSH2	c.G2245T	1	NA	NA	NA	P
32	45	Male	FLCN	c.1285dupC	1	5.39204E-05	4.823	0.6308–36.88	P
33	NA	Male	BRCA1	c.5407-2A>G	1	NA	NA	NA	P
34	42	Female	BRIP1	c.C2392T	1	0.000173402	1.5	0.2063–10.90	P
35	45	Male	PALB2	c.1059delA	1	NA	NA	NA	P
36	34	Male	MLH1	c.1377dupA	1	NA	NA	NA	P
37	42	Male	MSH6	c.C2731T	3	3.22893E-05	24.18	3.606–314.1	P
38	48	Male							
39	62	Male							
40	49	Female	RAD50	c.2980_2983del	1	4.47635E-05	5.81	0.7499–45.01	P
41	50	Female	APC	c.C3340T	1	NA	NA	NA	P
42	34	Female	APC	c.4014delG	1	NA	NA	NA	P
43	43	Female	MUTYH	c.G467A	5	5.71088E-05	22.79	8.206–63.31	P
44	60	Female							
45	48	Male							
46	57	Male							
47	52	Male							
48	65	Male	TP53	c.442+1G>A	1	0	192	7.821–4715	P
49	30	Female	APC	c.453-2A>T	1	NA	NA	NA	P
50	46	Male	BLM	c.319dupT	1	0.000012193	21.33	2.218–205.1	P
51	NA	NA	MLH1	c.208-1G>A	1	NA	NA	NA	P
52	66	Male	BRCA1	c.1039_1040del	1	NA	NA	NA	P
53	52	Female	MAX	c.359delA	1	NA	NA	NA	P
54	42	Male	PTEN	c.672dupA	1	NA	NA	NA	P
55	58	Male	MSH6	c.C2194T	1	4.07159E-06	63.88	3.995–1021	P
56	50	Female	MSH2	c.227_228del	1	NA	NA	NA	P
57	67	Female	BLM	c.295_296del	1	8.12691E-06	32	2.901–353.0	P
58	53	Male	BRCA1	c.G3196T	1	NA	NA	NA	P
59	50	Male	ATM	c.C7792T	1	8.17401E-06	31.82	2.884–351.0	P
60	63	Female	TP53	c.673-2A>G	1	NA	NA	NA	P
61	47	Male	MLH1	c.G677A	2	NA	NA	NA	P
62	43	Male							
63	48	Male	BRCA1	c.C4372T	1	NA	NA	NA	P
64	52	Male	APC	c.3955delC	1	NA	NA	NA	P
64	52	Male	TP53	c.G733A	1	0	192.1	7.825–4716	P
65	64	Female	RET	c.G1998C	1	NA	NA	NA	P
66	51	Male	PALB2	c.T2108G	1	8.12156E-06	32.02	2.903–353.2	P
67	71	Female	APC	c.4661dupA	1	NA	NA	NA	P
68	47	Female	PMS2	c.C1882T	1	1.62442E-05	16.01	1.789–143.3	P
69	38	Female	MSH2	c.A1648T	1	NA	NA	NA	P
70	70	Female	APC	c.C646T	1	4.08037E-06	63.74	3.986–1019	P
71	51	Male	MRE11A	c.1843-1G>T	1	NA	NA	NA	P
72	75	Male	RAD50	c.2498_2499del	1	4.06593E-05	6.396	0.8186–49.98	P
73	61	Male	PMS2	c.C943T	1	2.03169E-05	12.8	1.495–109.6	P
74	56	Male	BRCA1	c.C2599T	1	NA	NA	NA	P
75	57	Female	APC	c.C481T	1	NA	NA	NA	P
76	70	Male	ATM	c.8816_8826del	1	NA	NA	NA	P
77	63	Male	ATM	c.1402_1403del	1	4.06213E-05	6.402	0.8194–50.02	P
78	61	Male	BRCA1	c.981_982del	1	NA	NA	NA	P
79	46	Male	MSH2	c.630delG	1	NA	NA	NA	P
80	39	NA	RAD50	c.2157delA	2	0.000136161	3.821	0.9153–15.95	P
81	NA	NA							
81	NA	NA	AXIN2	c.1994delG	1	0	187.2	7.624–4595	P
82	38	Female	APC	c.3867_3871del	1	8.13643E-06	31.96	2.898–352.6	P
83	35	Female	APC	c.C2413T	1	NA	NA	NA	P
84	35	Male	MSH2	c.G1111T	1	NA	NA	NA	P
85	69	Male	ATM	c.7141_7151del	1	NA	NA	NA	P
				Overall		3.08701E-05	11.31	8.289–15.44	

*Data from gnomAD.

**Table 4:** Likely pathogenic germline mutations identified in this study

Patient ID	Age, years	Gender	Gene symbol	Nucleotide change	Allele count in this study	Allele frequency in the general population*	OR	95% CI	Annotation
1	73	Male	EPCAM	c.77-2A>G	1	4.09712E-06	63.48	3.970–1015	LP
9	52	Male	BRIP1	c.3072delG	2	8.12321E-06	64.05	9.020–454.8	LP
86	50	Female							
22	47	Male	APC	c.1743+1G>A	1	4.06484E-06	63.98	4.001–1023	LP
22	47	Male	MSH6	c.3254dupC	1	5.71447E-05	4.551	0.5983–34.62	LP
41	50	Female	TP53	c.G713A	1	8.12183E-06	.	2.903–353.2	LP
41	50	Female	APC	c.3921dupA	1	NA	NA	NA	LP
59	50	Male	MSH6	c.2740dupA	1	NA	NA	NA	LP
80	39	NA	PMS2	c.1145-1G>A	1	NA	NA	NA	LP
81	NA	NA	MSH2	c.C2271G	1	NA	NA	NA	LP
81	NA	NA	AXIN2	c.1212_1215del	1	5.49541E-06	47.33	2.960–756.8	LP
87	43	Female	MLH1	c.A250G	1	NA	NA	NA	LP
88	55	Male	MSH6	c.2554_2555del	1	NA	NA	NA	LP
89	54	Female	MSH2	c.943-2A>G	1	NA	NA	NA	LP
90	60	Female	RAD50	c.887delT	1	NA	NA	NA	LP
91	59	Male	ATM	c.1713delT	1	NA	NA	NA	LP
92	71	Female	RAD50	c.2976_2977del	1	4.06881E-06	63.92	3.997–1022	LP
93	45	Male	MLH1	c.G194A	1	NA	NA	NA	LP
94	55	Female	RAD50	c.C2476T	1	4.0659E-06	63.97	4.000–1023	LP
95	43	Female	MSH6	c.1866dupA	1	NA	NA	NA	LP
96	55	Male	MLH1	c.380+1G>T	1	NA	NA	NA	LP
97	NA	Male	NBN	c.1651delA	1	NA	NA	NA	LP
97	NA	Male	NTRK1	c.477_478insGC	2	NA	NA	NA	LP
98	NA	Male							
97	NA	Male	NTRK1	c.474_475del	2	NA	NA	NA	LP
98	NA	Male							
98	NA	Male	MSH6	c.G3725A	1	4.06583E-06	63.97	4.000–1023	LP
98	NA	Male	ATM	c.7411_7412insATTT	1	NA	NA	NA	LP
99	47	Male	ATM	c.T3900G	1	NA	NA	NA	LP
100	61	Male	MUTYH	c.C325T	1	3.65473E-05	7.116	0.9013–56.18	LP
101	48	Male	BLM	c.C3678A	1	NA	NA	NA	LP
102	45	Female	MLH1	c.345_349del	1	NA	NA	NA	LP
103	79	Female	SDHB	c.540+1G>A	1	NA	NA	NA	LP
104	49	Male	BLM	c.1440dupT	1	NA	NA	NA	LP
105	65	Female	MLH1	c.1612delT	1	NA	NA	NA	LP
105	65	Female	MLH1	c.1616_1619del	1	NA	NA	NA	LP
106	54	Male	BLM	c.371_372del	1	NA	NA	NA	LP
107	63	Female	BLM	c.3354delC	1	NA	NA	NA	LP
108	44	Male	BRIP1	c.C1471T	1	NA	NA	NA	LP
109	48	Female	MLH1	c.G794C	1	NA	NA	NA	LP
110	66	Female	ATM	c.7366_7367del	1	NA	NA	NA	LP
111	68	Male	MSH6	c.1698delA	1	NA	NA	NA	LP
112	44	Male	MSH6	c.994delG	1	NA	NA	NA	LP
113	46	Male	ATM	c.3609delT	1	NA	NA	NA	LP
114	75	Male	CHEK2	c.817_818del	1	4.07558E-06	63.81	3.991–1020	LP
115	49	Female	PALB2	c.1400delG	1	NA	NA	NA	LP
116	63	Male	NTRK1	c.1354+1G>T	3	1.62504E-05	48.04	10.75–214.7	LP
117	45	Female							
118	NA	Male							
119	63	Male	TP53	c.C817T	1	1.22115E-05	21.3	2.215–204.8	LP
120	64	Male	BLM	c.G2926T	1	4.06161E-06	64.03	4.004–1024	LP
121	43	Female	CHEK2	c.622delG	1	4.47579E-06	58.11	3.634–929.2	LP
122	51	Male	PMS2	c.1144+1G>A	1	8.13107E-06	31.99	2.900–352.8	LP
123	43	Female	MSH2	c.1510+1G>A	1	4.06303E-06	64.01	4.003–1024	LP
124	59	Female	MLH1	c.1990_1997del	1	NA	NA	NA	LP
125	26	Female	APC	c.1908dupT	1	NA	NA	NA	LP
126	54	Female	MSH2	c.G2021A	1	NA	NA	NA	LP
127	62	Male	PMS2	c.803+1G>A	1	NA	NA	NA	LP
128	71	Male	NF1	c.3198-1G>T	1	NA	NA	NA	LP
129	47	Female	RAD51D	c.271_272insTA	1	5.68574E-05	4.574	0.6013–34.79	LP
130	29	Female	MSH2	c.838_839insTG	1	NA	NA	NA	LP
131	63	Male	EXT2	c.C174G	1	NA	NA	NA	LP
132	43	Female	MSH2	c.175delA	1	NA	NA	NA	LP
133	45	Male	MSH2	c.G2074A	1	NA	NA	NA	LP
134	41	Male	MLH1	c.G2041A	1	NA	NA	NA	LP
135	55	Male	RAD51D	c.C898T	1	0.000028437	9.145	1.125–74.35	LP
136	48	Male	MSH2	c.1602dupT	1	NA	NA	NA	LP
137	52	Female	TP53	c.T737C	1	NA	NA	NA	LP
138	45	Female	RAD51D	c.627dup	1	NA	NA	NA	LP
139	42	Female	MSH2	c.T1764A	1	NA	NA	NA	LP
140	42	Male	SDHA	c.1064+2T>C	1	NA	NA	NA	LP
141	55	male	RAD50	c.134delT	1	NA	NA	NA	LP
142	43	Male	CHEK2	c.1375+2T>A	1	NA	NA	NA	LP
143	55	Female	ATM	c.6129dupC	1	NA	NA	NA	LP
144	35	Male	SDHA	c.A1G	1	8.56663E-06	30.36	1.899–485.5	LP
145	39	Male	MLH1	c.16delG	1	NA	NA	NA	LP
146	NA	NA	MRE11A	c.929_930insTGATTAGCTAGAA CAATATCCTCCATGAAAAACTGC CGCACTGTGTGAAGAGGAATTT TATGCATATTCATCTTCACACAGT GCGGCAGTTTTTCATGGA	1	NA	NA	NA	LP
147	44	Female	MSH2	c.646-2A>G	1	NA	NA	NA	LP
				Overall		1.44636E-05	20.68	12.89–33.18	

*Data from gnomAD.

Some patients with CRC recruited for this study lacked prognostic data. Consequently, we were unable to perform prognostic analysis. However, prognostic data were successfully obtained from a previous report^11^; the patient prognosis was then compared between those with and without germline mutations. As shown in **Supplementary Figure S2**, patients with germline mutations exhibited significantly poorer overall survival than those without germline mutations (*P* = 0.0087). The median survival time for the germline group was 1,323 days, whereas the median survival for the non-germline group had not been reached.

## Discussion

Previous research has identified correlations between P germline mutations and hereditary CRC, including MLH1/MSH2/MSH6/PMS2 mutations with Lynch syndrome (also known as hereditary non-polyposis CRC), APC mutations with FAP, MUTYH mutations with MUTYH-associated polyposis, STK11 mutations with Peutz-Jeghers syndrome, SMAD4/BMPR1A mutations with juvenile polyposis syndrome, PTEN mutations with PTEN hamartoma tumor syndrome, and RNF43 mutations with serrated polyposis syndrome^4–7^. Although the relationships among these diseases and mutations are known, the frequency, location, and distribution of germline mutations in the Chinese population, and their quantitative relationships with CRC risk have yet to be elucidated. The distribution of rare germline mutations and their roles in the pathogenesis of CRC are also worthy of exploration. In addition, no systematic studies have investigated the similarities and differences in the somatic mutational landscape between patients with and without P/LP germline mutations. In this study, we recruited a large cohort of 1,923 cases and systematically investigated germline mutations and corresponding somatic mutational alterations in a Chinese population.

As expected, a significantly higher proportion of patients with P or LP mutations had a family history of CRC than did non-P patients, thus suggesting that these germline mutations increased the risk of CRC in affected families. Because of the high proportion of affected MMR genes in P and LP mutations, the proportion of patients with dMMR and MSI-H was significantly higher in these groups; therefore, these patients may respond well to immunotherapy. Our results also confirmed the early onset of CRC in patients with P or LP mutations, thereby indicating a similar trend to those of FAP and Lynch syndrome. Although some novel mutations were not determined to be pathogenic, their overall influence appeared to be similar to that of confirmed hereditary CRC.

We found that 7.6% of patients (147/1,923) carried P/LP mutations, and 1.4% of patients (27/1,923) had Lynch syndrome; these findings are similar to the proportions previously published for both Chinese and Western populations^7,12,13^. However, because of a lack of sufficient evidence for LP germline mutations, many mutations in MLH1, MSH2, MSH6, and PSM2 could not be confirmed as Lynch syndrome mutations. Therefore, the incidence of Lynch syndrome might have been underestimated, and the actual incidence could have exceeded 2%, as described in previous reports^7,12,13^. The APC gene had the highest number of P germline mutations, thus indicating that FAP is the most common form of hereditary CRC in Chinese population, followed by Lynch syndrome. In addition, ATM gene germline mutations have been detected in other malignant tumors^14^. Because ATM is an important candidate member of the DNA damage and repair (DDR) pathway, germline mutations may directly lead to abnormal DNA repair. The present evidence suggests that ATM germline mutations are not cancer type-specific, because they have been reported in many cancers and have been suggested to potentially increase the risk of some cancers^14^. In the present study, the OR of P ATM mutations varied from 6.4 to 63.87, thus suggesting an increased risk in patients with CRC carrying these mutations. We also identified several BRCA1 and BRCA2 germline mutations in this study. BRCA1/2 genes, encoding products that participate in the DDR and HRR pathways, represent confirmed carcinogenesis of hereditary breast and ovarian cancer syndrome. BRCA1/2 germline mutations have also been reported in CRC^15^. All BRCA1/2 P germline mutations reported herein are associated with CRC, on the basis of clear clinical evidence. Our previous studies have also confirmed that BRCA2 germline mutations increase the risk of lung cancer^10^. Because no hotspot mutations have been reported in BRCA1/2 in the Chinese population, many mutations were categorized as LP or VUS. Additional clinical evidence is necessary to confirm their pathogenicity in cancer.

We compared the ratio and distribution of germline mutations between Chinese and Western populations by using the data from the present study and data reported by Hahnen et al.^11^ in 2017. We found that PALB2 was ranked as the top P mutation in the Western population but had a much lower ranking in the Chinese population (**Supplementary Figure S3A**). In contrast, APC was ranked as the top P mutation in the Chinese population but was not detected in the Western population. Moreover, ATR was ranked as the top LP mutation in the Western population but was not detected in the Chinese population. Differences between these populations were also reflected in the proportion of patients with Lynch syndrome. The proportion of patients with Lynch syndrome with P mutations in the Chinese population was 29.3% (27/92), compared with a ratio of 15.0% in the Western population (3/20) (**Supplementary Figure S3B**). These comparisons indicate a potential differential germline mutational landscape in CRC.

Frameshift and nonsense mutations were the 2 most common types of mutations detected in the study, followed by missense and splicing mutations. Frameshift and nonsense mutations lead to the partial or complete loss of function of corresponding proteins, thus increasing the risk of cancer in mutation carriers. Missense mutations in key amino acids can also induce substantial changes in protein function, whereas splicing mutations can influence transcription and subsequent translation. We found that most mutations in highly mutated genes were located in known functional domains, thus reflecting the roles of these domains in maintaining normal protein function. Indeed, because all mutations identified in this study were heterozygous, a partial loss of function might be compensated for by the other normal allele. These heterozygous mutations might not be lethal but could increase the risk of cellular aberrant transformation and carcinogenesis.

In this study, we conducted the first comparative study of somatic mutational landscapes on the basis of the pathogenicity classification of germline mutations. We found that the mutational frequency of most of the highly mutated genes in the P group was higher than that in the non-P group; the LP group also showed a similar trend toward a higher mutational frequency, possibly because the mutations in the P group affected the MMR, DDR, and homologous recombination deficiency pathways, thus leading to abnormal DNA repair and a large number of somatic mutations^16^. The patients with and without Lynch syndrome in the P group showed a similar trend, and the mutational frequency in patients with Lynch syndrome was much higher than that in patients without Lynch syndrome. This finding was also confirmed by TMB statistics: the TMB of patients with Lynch syndrome was significantly higher than that of the other 3 groups. TMB has been suggested to be an effective indicator for patient prognosis stratification in immunotherapy^17^. Our data provided strong evidence supporting the use of immunotherapy in patients with Lynch syndrome. Interestingly, we observed no difference in the frequency of APC and KRAS mutations across the 3 groups, thus suggesting that major driver gene mutations may be common driving factors for CRC, regardless of P germline mutations. In addition, our data showed that the CNV variation in the non-P group was higher than that in the P group, and that CNV variation in the patients without Lynch syndrome was also higher than that in patients with Lynch syndrome, thus indicating a seesaw effect. That is, a higher proportion of SNV/indel mutations corresponded to a lower proportion of CNV alterations, whereas a lower proportion of SNV/indel mutations corresponded to a higher proportion of CNV alterations. This observation suggests that CRC is a highly heterogeneous cancer in which pathogenesis is diverse and depends on different types of genetic alterations. The co-existence and balance of mutations and CNVs may be related to both genetic and environmental backgrounds. Similar observations of the seesaw effect have also been reported in other studies^10,18,19^.

Our detailed clustering analysis led to interesting discoveries. We found the first reported evidence that the Notch pathway is clustered in only patients with Lynch syndrome with P germline mutations, but not patients without Lynch syndrome. Furthermore, we observed that the MAPK and cAMP signaling pathways were clustered in patients without Lynch syndrome but not patients with Lynch syndrome. In contrast, the Wnt and calcium signaling pathways, along with the human papillomavirus infection pathway, were all clustered in CRC. This finding suggests that the Notch pathway is specific to patients with Lynch syndrome, whereas the MAPK and cAMP signaling pathways are specific to patients without Lynch syndrome. The Wnt and calcium signaling pathways, along with human papilloma virus infection, may be common pathogenic factors for CRC, regardless of germline mutations. The Notch pathway plays an important role in embryonic development, cell proliferation, and differentiation. Furthermore, the role of the Notch pathway has been investigated for many different types of tumors^20^, including CRC^21^. However, the role of the Notch pathway in Lynch syndrome has not been studied previously. Our identification of Lynch-specific Notch pathway activity demonstrated the existence of distinct pathogenic mechanisms in patients with Lynch syndrome and patients without Lynch syndrome with CRC; therefore, our research provides key information that may facilitate molecular typing.

In this study, we report the first quantification of the risk of CRC associated with P and LP germline mutations. We also calculated the overall OR for the P and LP groups. The frequency of mutations identified by gnomAD screening represents the frequency of a certain alteration in the general population. Because most P or LP germline mutations exhibited very low incidence, the frequency in the general population, and in patients with cancer, may exhibit a certain degree of randomness and may not accurately represent the true frequency. Thus, the overall OR for the P or LP group as a whole may have greater relevance and significance for the population. For some relatively common germline mutations, such as those from APC and the 4 MMR genes, the risk associated with individual genes can be calculated; for the less frequent gene mutations, larger population studies and familial evidence are urgently needed. In this study, the overall OR of both the P and LP groups exceeded 10, thus suggesting that patients with such germline mutations had a significantly greater risk of CRC than the average-risk population. Previous studies of other cancers also support this method for evaluating the risk of germline mutations from population data^10,22,23^. From the perspective of treatment, personalized therapeutic strategies should be given to patients with such mutations, and more frequent and detailed examinations should be performed on their unaffected family members carrying these mutations. This practice would enable detection of tumors as early as possible and support early intervention.

## Conclusions

In this study, we fully characterized germline and somatic mutations in Chinese patients with CRC. We found that 7.6% of our study cohort carried germline variants linked to greater susceptibility to CRC. Patients with P or LP mutations had a higher proportion of MSI-H, dMMR, family history of CRC, and significantly lower age. The somatic mutations in Chinese patients with patients with CRC were fully characterized and found to exhibit distinct features. The Notch signaling pathway was uniquely clustered in patients with Lynch syndrome, whereas the MAPK and cAMP signaling pathways were uniquely clustered in patients with CRC who did not have Lynch syndrome. Our findings provide important information for potential molecular typing and therapy for patients with CRC with germline mutations.
