# Supplementary material for: Comprehensive characterization of CRC with germline mutations reveals a distinct somatic mutational landscape and elevated cancer risk in the Chinese population
Source: Cancer Biol Med. 2022 Jan 12;19(5):707–32. doi: 10.20892/j.issn.2095-3941.2021.0190 (PMC9196063; doi:10.20892/j.issn.2095-3941.2021.0190)
Supplement: Supplementary file 1 [file cbm-19-707-s001.pdf]

Supplementary materials

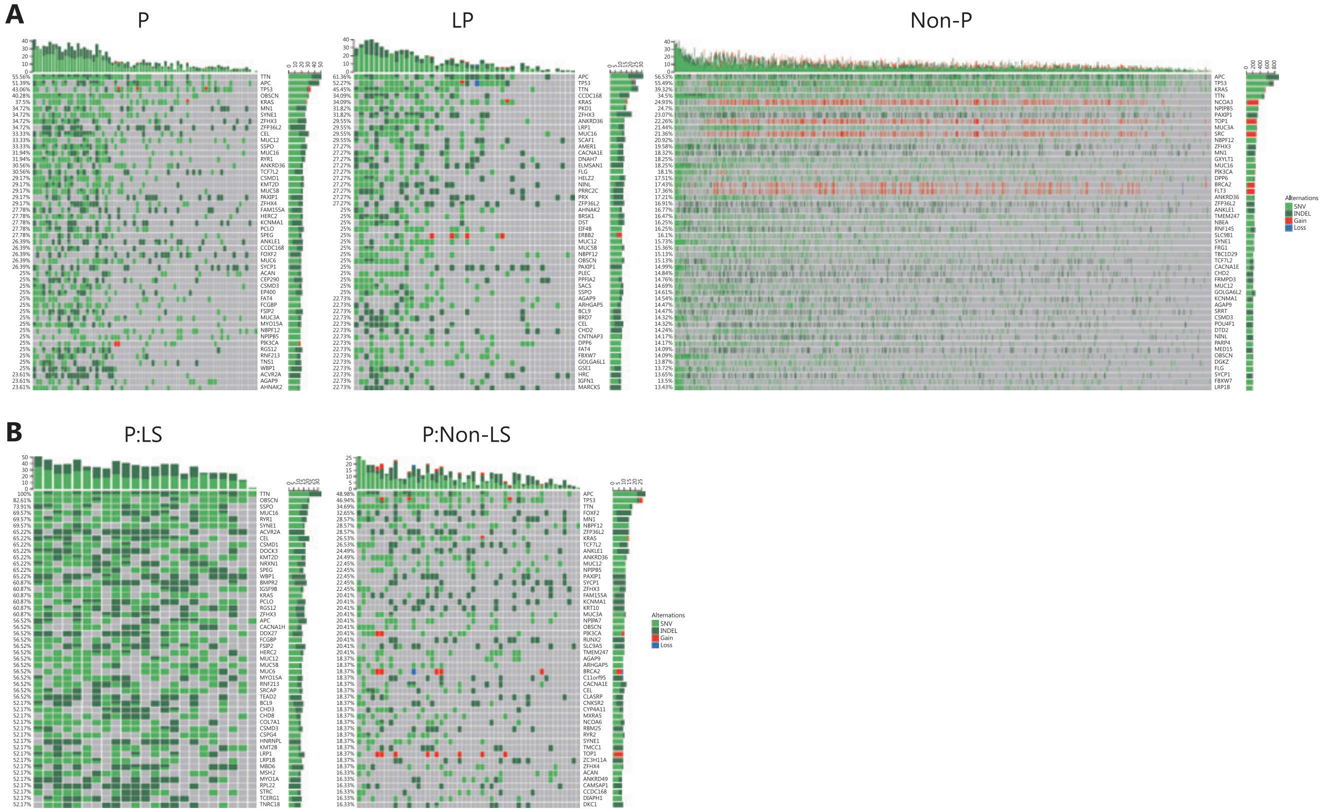

Figure S1 Mutational landscape of patients with CRC in this study. A. The mutational landscapes of patients with CRC in the P, LP, and non-P groups. B. The mutational landscapes of patients with Lynch syndrome (LS) or without Lynch syndrome (non-LS) in the pathogenic (P) group.

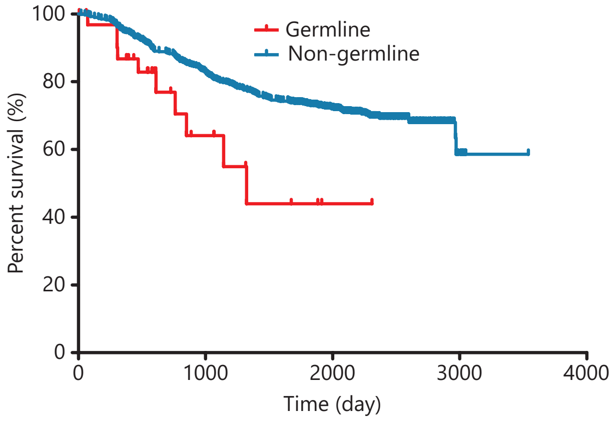

Figure S2 Kaplan-Meier analysis of overall survival of patients with CRC with or without germline mutations. A significant difference was observed in overall survival between groups (P = 0.0087). The median survival was 1,323 days for the germline group and was not reached in the non-germline groups.

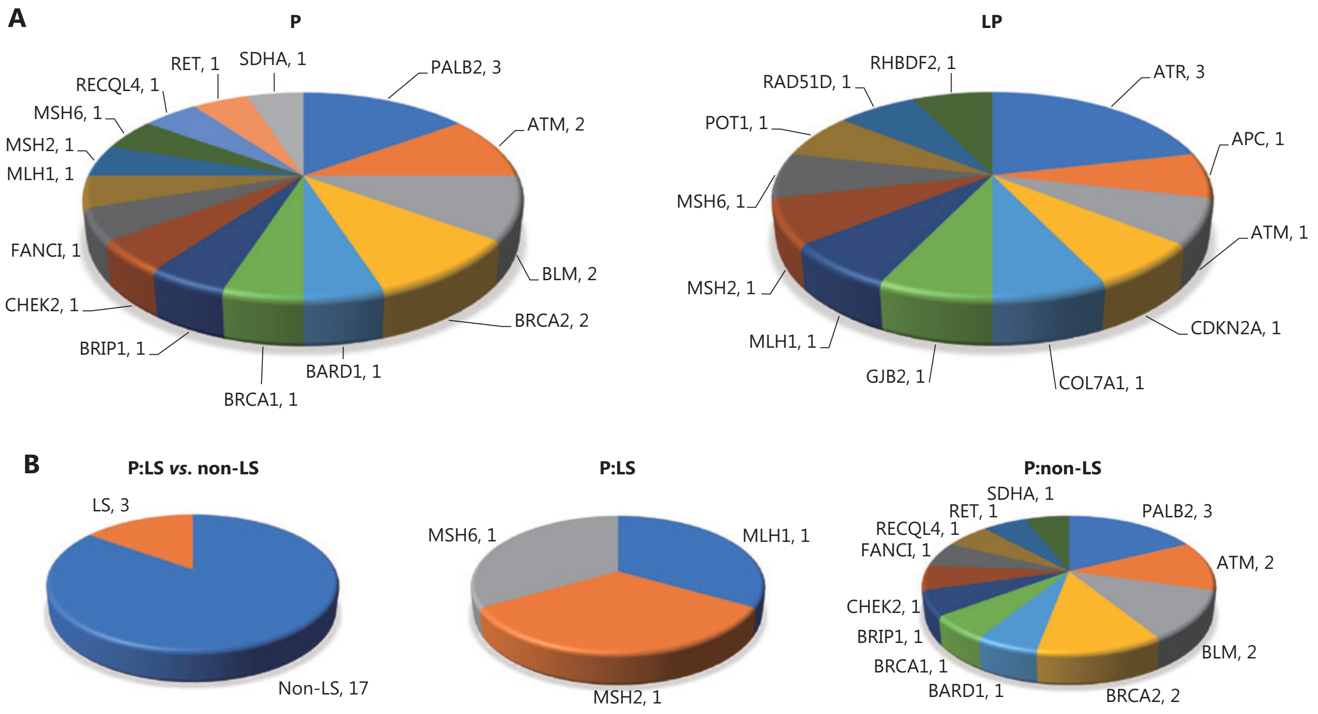

Figure S3 Category and distribution of germline mutations from data in reference 11 (Western population). A. The number of mutations in highly mutated genes in the pathogenic (P) and likely pathogenic (LP) groups. B. Details of mutated genes and their numbers in the Lynch syndrome (LS) and non-Lynch syndrome (non-LS) groups.
